# Acupuncture for patients with premature ovarian insufficiency: A systematic review protocol

**DOI:** 10.1097/MD.0000000000015444

**Published:** 2019-05-03

**Authors:** Li Huang, Yu Chen, Mei Luo, Yancai Tang, Shaobin Wei

**Affiliations:** a College of Clinical Medicine, Chengdu University of Traditional Chinese Medicine; b Department of Gynecology, Hospital of Chengdu University of Traditional Chinese Medicine, Chengdu, Sichuan, China.

**Keywords:** acupuncture, premature ovarian insufficiency, protocol, systematic review

## Abstract

**Background:**

Premature ovarian insufficiency (POI) is a difficult-to-treat gynecological disorder with complex etiologies. Although acupuncture has gained increased popularity for the management of POI, evidence regarding its efficacy is lacking. This systematic review protocol aims to describe a meta-analysis to assess the effectiveness and safety of acupuncture for patients with POI.

**Methods:**

The following 10 databases will be searched from the publishment to July 2019: PubMed, Embase, the Web of Science, the Cochrane Central Register of Controlled Trials, 4 Chinese databases (China National Knowledge Infrastructure, Wanfang Digital Periodicals, Chinese Biomedical Literature Database, Chinese Scientific Journal Database database), 1 Korean medical database (KoreaMed), 1 Japanese medical database (National Institute of Informatics). The primary outcomes will be the resumption of menstruation and the serum FSH levels, and the secondary outcomes include the serum Estradiol levels, anti-Mullerian hormone levels, antral follicle count, follicular growth, endometrial thickness, and adverse events. We will use RevMan V.5.3 to conduct the meta-analysis, if possible. If it is not allowed, a descriptive analysis or a subgroup analysis will be conducted. Risk ratio for dichotomous data and mean differences or standardized mean differences for continuous data will be calculated with 95% confidence intervals using a random effects model or a fixed effects model.

**Results:**

This study will provide the latest analysis of the currently available evidence for the efficacy of acupuncture in treating POI.

**PROSPERO registration number:**

CRD42019125996.

## 1 Introduction

Premature ovarian insufficiency (POI) refers to a gynecological endocrine disease characterized by oligomenorrhea or amenorrhea, hypoestrogenism, elevated serum gonadotropin levels before the age of 40 years.^[1]^ The consequences of POI are estrogen deficiency, infertility, reduced bone mineral density, cardiovascular disease, genito-urinary symptoms, and cognitive impairment.^[2]^ Given the long-term health consequences, POI has a significant negative impact on psychological health and quality of life. The prevalence of POI is estimated to be 1%.^[3]^ Epidemiological studies have shown differences in the occurrence of POI depending on demographic characteristics. A recent national register study including 1,036,918 women from Sweden showed the total prevalence of POI was 1.9%.^[4]^A pooled study including 51,450 postmenopausal women from 9 observational studies in UK, Scandinavia, Australia, Japan, showed a prevalence of premature menopause of 2%,^[5]^ indicating a higher prevalence than previously reported. The etiopathology of POI remains unknown. Recognized causes of POI include genetic aberrations, metabolism disorders, autoimmunity, iatrogenic factors, infections, toxins, and environmental factors.^[6–8]^

Although there is no definite and effective way to restore ovarian function, hormone replacement therapy (HRT) remains an appropriate treatment for women with symptoms of estrogen deficiency. It is widely considered that HRT may play a role in primary prevention of cardiovascular system disease and bone protection.^[1]^ However, the risk of breast cancer is concerned. Previous studies assessing the safety of HRT in the population of postmenopausal women cannot be simply applied to women with POI.^[9]^

Acupuncture, a common traditional Chinese medicine (TCM) treatment modality, has been extensively used by clinicians for the management of POI. Clinical observations suggested that acupuncture can be potentially used to increase the efficient/response rate of clinical treatment of POI, improve menstrual conditions, reduce serum FSH and LH levels, and increase serum estradiol levels.^[10–13]^ Besides, acupuncture has few adverse effects such as vaginal bleeding, acute liver damage and liver dysfunction, vascular embolization. There are some possible mechanisms of acupuncture. In TCM theory, acupuncture is considered to function by regulating Qi and Blood, replenishing kidney essence, coordinating thoroughfare and controlling vessels.^[14]^ Although the mechanism of acupuncture for POI has not been fully investigated, studies in animals signified that acupuncture could modulate the hypothalamic-pituitary-ovary axis and IGF-1/IGF-1R axis,^[15,16]^ elevate estrogen receptor expression,^[17]^ and regulate Bcl-2/Bax levels.^[18]^

A systematic review of acupoint stimulation therapy for POI in 2017 involved researches on various acupoint stimulation therapies, such as acupuncture, moxibustion, electroacupuncture, ear point, tuina, cupping, point catgut-embedding therapy, transcutaneous electrical stimulation at acupoint.^[19]^ The outcomes were effective rate and adverse effect, which were not clearly described. Besides, the study failed to evaluate the safety of each acupoint stimulation therapy. Acupoint stimulation therapy is widely used in Asia, but only 3 English databases and 4 Chinese databases were searched in this study. Therefore, this review had some flaws that threatened the authenticity of their findings. Several systematic reviews about the effectiveness of acupuncture in premature ovarian failure (POF) have been published.^[20,21]^ Guideline on the management of women with POI was published in 2016, and the diagnosis of POI was different from POF. Whether the evidence is transferable to POI remains unclear. Overall, no definite conclusions on the effectiveness of acupuncture for POI can be drawn.

The present systematic review aims to assess the efficacy and safety of acupuncture compared with HRT, sham acupuncture, and no treatment. This systematic review will provide convincing conclusions by using a strict search strategy and an objective outcome evaluation.

## 2 Methods

### 2.1 Study registration

This systematic review protocol has been registered in the International Prospective Register of Systematic Reviews (PROSPERO) as CRD42019125996. The protocol is conducted complying with the preferred reporting items for systematic reviews and meta-analysis protocols (PRISMA-P) statement guidelines.^[22]^ And the study will follow the PRISMA statement guidelines.^[23]^

### 2.2 Criteria for including studies

#### 2.2.1 Types of studies

All randomized controlled trials (RCTs) without restrictions will be included in this review. We will also include summary results of completed and ongoing trials published on clinical trial registration platform. Quasi-randomized controlled studies will be excluded. Studies with a sample size of less than 20 patients will be excluded.

#### 2.2.2 Types of participants

Patients diagnosed with POI according to management of women with POI^[1]^ and expert consensus on HRT for POI^[8]^ will be included. The diagnostic criteria for POI include age <40 years, oligo/amenorrhea for at least 4 months, and an elevated FSH level >25 IU/L on 2 occasions >4 weeks apart.^[1]^ There will be no restrictions on ethnicity, nationality, education, or economic status.

#### 2.2.3 Types of interventions and comparisons

We will include trials using any type of acupuncture regardless of frequency, intensity, duration. Acupuncture refers to the stimulation of acupoint by needles, such as manual acupuncture, electroacupuncture, warm needling, fire needling, body acupuncture, dermal needle, auricular acupuncture, scalp acupuncture, and plum blossom needle. Trials evaluating acupuncture as a combination of HRT will also be included. We will exclude the trails in which patients were treated with other stimulating methods, such as acupressure, moxibustion, acupoint injection, laser acupuncture, and cupping.

Trials involving HRT, sham acupuncture, no treatment for a control group will be considered. Studies comparing the efficacy of different acupoint prescriptions or other complementary and alternative therapeutic intervention (eg, Chinese herbal medicine) will be excluded. The following treatment comparisons will be considered.

1. Acupuncture versus HRT.
2. Acupuncture versus sham acupuncture.
3. Acupuncture versus no treatment.
4. Acupuncture plus HRT versus HRT alone.

#### 2.2.4 Types of outcome measures

##### 2.2.4.1 Primary outcome measures

The primary outcome will be the resumption of menstruation and the serum FSH levels between day 2 to day 5 of the menstrual cycle. The resumption of menstruation is defined as the return of menses in patients with amenorrhea for at least 4 months.

##### 2.2.4.2 Secondary outcome measures

1. The serum estradiol levels between day 2 to day 5 of the menstrual cycle.
2. Anti-Mullerian hormone levels.
3. Antral follicle count between day 2 to day 5 of the menstrual cycle (assessed by transvaginal ultrasound).
4. Follicular growth (growth of the follicle to size at least 18 mm monitored by transvaginal ultrasound).
5. Endometrial thickness (endometrial thickness in women with thin endometrium (less than 8 mm) monitored by transvaginal ultrasound).
6. Adverse events.

### 2.3 Search strategy

#### 2.3.1 Electronic searches

We will electronically search the following databases from their inception to July 2019: the Cochrane Central Register of Controlled Trials, PubMed, Embase, the Web of Science, China National Knowledge Infrastructure, Wanfang Digital Periodicals, Chinese Biomedical Literature Database, Chinese Scientific Journal Database, KoreaMed, National Institute of Informatics. Any clinical RCTs related to acupuncture for treating POI without restriction of publication status and languages will be included.

The search strategies for PubMed are summarized in Table 1. These search terms will be precisely translated for other databases.

**Table 1:**
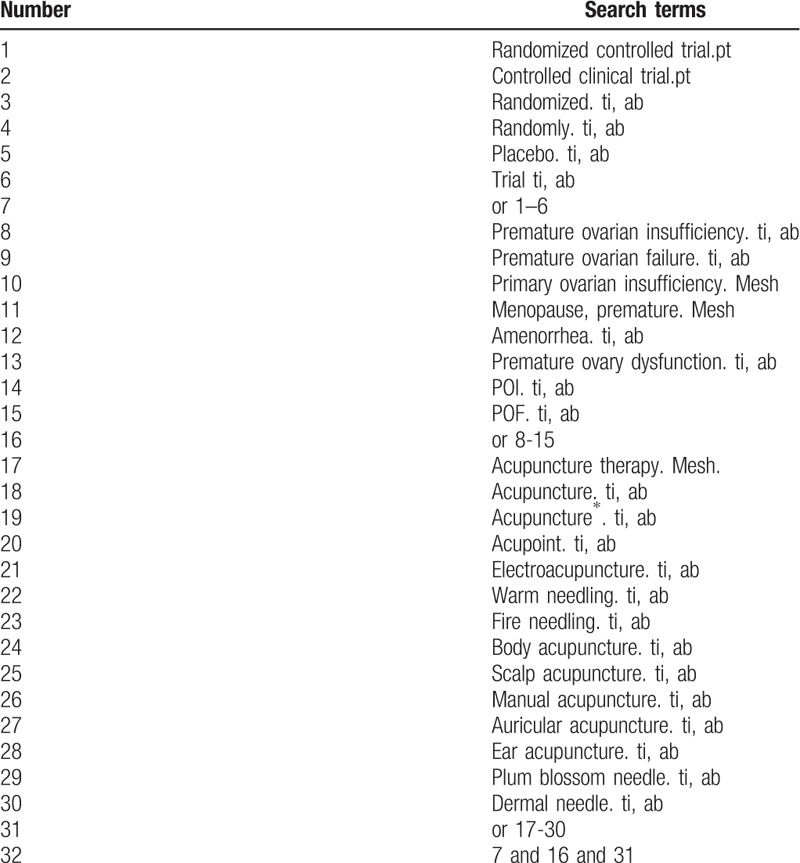
Search strategy for PubMed.

#### 2.3.2 Searching other resources

We will review the reference lists of relevant RCTs and meta-analysis for potentially eligible studies. We will also search ongoing or unpublished trials from the NIH Clinical Trails (https://www.clinicaltrials.gov/), the International Clinical Trials Registry Platform (http://www.who.int/ictrp/), and the Chinese Clinical Register (http://www.chictr.org/).

### 2.4 Data collection and analysis

#### 2.4.1 Selection of studies

**Figure 1:**
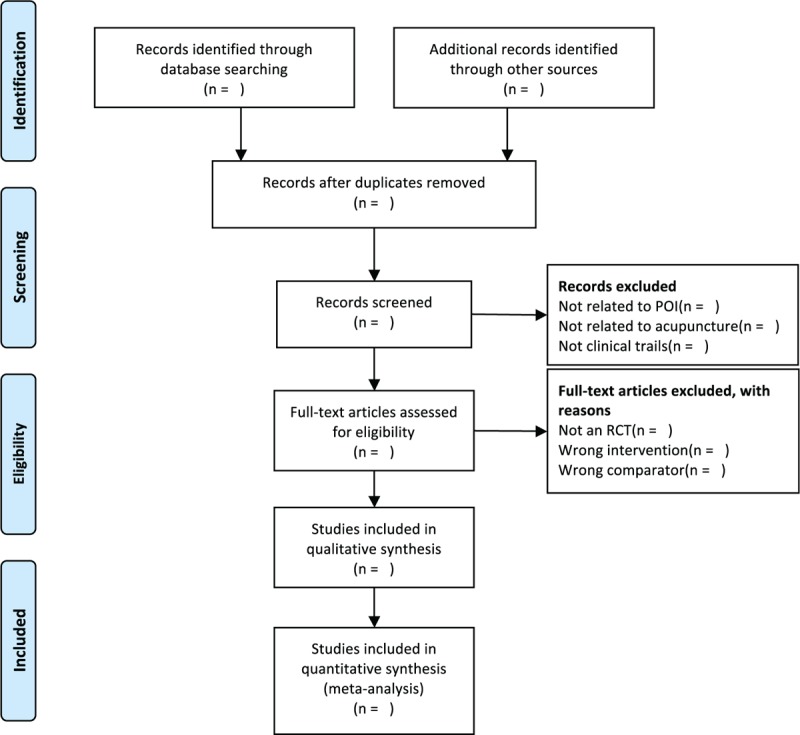
The PRISMA flow diagram of the study selection process. PRISMA = preferred reporting items for systematic reviews and meta-analysis protocols.

We will use EndNote software (V.X7.0) to omit duplicates and manage records. Two independent reviewers (YC and ML) will check titles and abstracts of studies according to the inclusion criteria. Reviewers will obtain full-text reports for further assessment. The explanations for exclusion will be recorded in an excel data set. Disagreements will be resolved by discussion. If necessary, further argument will be arbitrated by a third reviewer (LH). The study flow diagram is shown in Figure 1.

#### 2.4.2 Data extraction and management

Two independent reviewers (LH and YCT) will double check the eligibility of the included studies and extract data using a predefined data acquisition form. This form will include 4 main domains: general information (title, authors, country of study, funding, journal, year of publication); details of study methods (design, sample size, method of randomization, allocation concealment, blinding); participant characteristics (age, diagnostic criteria, disease duration); intervention details (type of acupuncture/control, treatment duration, treatment frequency); outcomes (primary and secondary outcomes, method of outcome assessments, time points). We will try to contact the corresponding author for any missing data or unclear information.

#### 2.4.3 Assessment of risk of bias in included studies

The methodological quality for each included trial will be evaluated using the Cochrane Collaboration's tool.^[24]^ Two reviewers (YC and LH) will independently evaluate the risk of bias. The discrepancy will be discussed to reach an agreement. If necessary, the third review author (ML) will be consulted. Risk of bias assessment categories will include the following 7 domains:

(1) randomized sequence generation;
(2) allocation concealment;
(3) blinding of participants;
(4) blinding of outcome assessors;
(5) incomplete outcome data;
(6) selective outcome reporting;
(7) other biases.

For each domain, the risk of bias will be evaluated as low, high, or unclear. We will contact the corresponding author if the basic information is missing about the risk of bias assessment.

#### 2.4.4 Measures of treatment effect

We will use RevMan V.5.3 for quantitative data synthesis and data analysis. For dichotomous data, we will use the risk ratio with 95% confidence intervals (CIs) to assess the treatment effect. For continuous data, we will use the standardized mean difference with 95% CIs when calculating the same outcome variables using different methods. We will use the mean difference with 95% CIs to estimate a treatment effect when the same outcome scale or method is used.

#### 2.4.5 Dealing with missing data

If there is missing or insufficient data, we will try to contact the corresponding author or the first author. An intention-to-treat analysis that includes all randomized patients will be conducted. For patients with missing outcome data, last observation carry-forward analysis or extreme worst-case analysis will be performed.^[25]^ We will use a sensitivity analysis to determine whether the results are inconsistent.

#### 2.4.6 Assessment of heterogeneity

Heterogeneity will be assessed by the *I*^2^ and *χ*^2^ tests. When the *I*^2^ value ≤50%, the study will be considered to have no heterogeneity. *I*^2^ value >50% will indicate the presence of substantial heterogeneity in the included studies.^[26]^ Potential clinical heterogeneity will be investigated by subgroup analysis. If *I*^2^ value >75%, a meta-analysis will not be conducted.^[27]^ Instead, we will qualitatively describe the effectiveness and safety of acupuncture.

#### 2.4.7 Assessment of reporting biases

If the numbers of studies included in the meta-analysis are sufficient (≥10 trials), we will use funnel plots to detect the reporting bias.^[28]^ An Egger test will be conducted to evaluate the funnel plot asymmetry. Since the funnel plot asymmetry does not necessarily suggest publication bias, we will try to analyze possible factors, such as poor methodological quality and small-study effects.

#### 2.4.8 Data synthesis

Meta-analysis will be performed using RevMan V.5.3. Clinical data will be synthesized and analyzed according to the level of *I*^2^ value. If *I*^2^ value ≤50%, we will use a fixed effect model. Instead, a random effect model will be used.^[29]^ If *I*^2^ value >75%, we will present a descriptive analysis with the effect size and 95% CI of each clinical trial.

#### 2.4.9 Subgroup analysis

A subgroup analysis will be conducted to explore the potential causes of heterogeneity if necessary. The following subgroup analysis plan will be considered.

1. Different types of intervention forms (acupuncture alone or adjunctive to HRT).
2. Different types of acupuncture (manual acupuncture, electroacupuncture, warm needling, etc).
3. Different types of the control group (HRT, sham acupuncture, no treatment).
4. Duration of follow-up (1–3 months, up to 6 months, more than 6 months).

#### 2.4.10 Sensitivity analysis

To identify the robustness of the primary results, we will conduct a sensitivity analysis by removing low-quality articles. Sample size and the effect of the missing data will be taken into account.

#### 2.4.11 Grading the quality of evidence

The quality of evidence for all outcomes will be evaluated by the Grading of Recommendations Assessment, Development, and Evaluation software. We will consider the following domains: risk of bias, consistency, directness, precision, publication bias, and additional points. The quality of evidence will be categorized as high, moderate, low, and very low.

## 3 Discussion

Many clinical observations have confirmed the effectiveness and safety of acupuncture in relieving the symptoms of POI. Besides, Chinese experts have agreed that acupuncture can be used as an adjunct or temporary alternative treatment therapy in the treatment of POI.^[30]^ Nevertheless, there is no systematic review for comparison with HRT, which adopts the latest diagnostic criteria according to *ESHER Guideline: management of women with premature ovarian insufficiency.* Therefore, we conduct this systematic review and meta-analysis to assess the effectiveness and safety of acupuncture for patients with POI. There are some potential limitations in this systematic review. First, different types of acupuncture therapies may run the risk of heterogeneity. Second, acquiring the complete raw data from original trials is difficult. If we cannot fully obtain the raw data, we will report the details and the results of possible bias. We hope this study will provide the latest analysis of the currently aggregated evidence for the efficacy of acupuncture in treating POI, which will benefit practitioners, patients, and healthcare policymakers.

## Author contributions

**Investigation:** Li Huang, Shaobin Wei.

**Methodology:** Mei Luo.

**Resources:** Li Huang, Yu Chen, Mei Luo, Yancai Tang.

**Supervision:** Shaobin Wei.

**Writing – original draft:** Li Huang.

**Writing – review and editing:** Shaobin Wei.
